# The Definitive‐Intent 10‐Fraction Radiotherapy Protocol (‘The Zurich Protocol’) Yields Lasting Tumour Control and Survival in Dogs With Intracranial Meningiomas

**DOI:** 10.1111/vco.70081

**Published:** 2026-06-16

**Authors:** Carlotta Ahrens, Sergejs Unterkirhers, Carla Rohrer Bley

**Affiliations:** ^1^ Clinic for Radiation Oncology & Medical Oncology, University Animal Hospital, Vetsuisse Faculty University of Zurich Zurich Switzerland; ^2^ Department of Physics University of Zurich Zurich Switzerland; ^3^ Radiotherapie Hirslanden AG Aarau Switzerland

**Keywords:** brain tumour, dog, dose‐volume constraints, meningioma, radiation‐induced toxicity

## Abstract

Moderately hypofractionated definitive‐intent radiotherapy protocols can be used for canine intracranial meningiomas to shorten overall treatment time while maintaining tumour control and limiting normal tissue toxicity. This retrospective study evaluated oncologic outcomes, toxicity, and radiation dose‐volume‐related prognostic factors in dogs with imaging‐based intracranial meningiomas treated with a 10‐fraction definitive‐intent protocol. Medical records from 2015 to 2025 were reviewed. All dogs received 10 daily fractions of 4 Gy delivered with a homogeneous dose prescription, risk‐adapted heterogeneous dose distributions, or an 11% simultaneously integrated boost (SIB) to the gross tumour volume. One hundred and ten dogs were included, with a median follow‐up of 498 days. Median time to progression was 873 days (95% CI: 552–1195), median overall survival after first radiotherapy was 756 days (95% CI: 474–1039), and median DSS was 1078 days (95% CI: 915–1242). Time to progression differed among treatment subgroups (*p* = 0.024). Larger volumes of normal brain receiving ≥ 32 Gy (> 11.5 cm^3^) were associated with shorter overall (*p* < 0.001) and disease‐specific survival (*p* = 0.002). Use of an SIB was associated with shorter disease‐specific survival (HR 2.47; *p* = 0.012), and post‐radiotherapy seizures were associated with reduced time to progression (HR 2.01; *p* = 0.026). Tumour location was not identified as a prognostic factor. Acute or early delayed neurologic toxicity occurred in 10% of dogs and was generally transient and generally corticosteroid responsive. In conclusion, the definitive‐intent, moderately hypofractionated 10‐fraction radiotherapy protocol achieves long lasting tumour control and survival outcomes in dogs with intracranial meningiomas, provided that evidence‐based brain dose‐volume constraints are respected.

## 1 Introduction

Fractionated radiotherapy remains a standard treatment for canine intracranial tumours, including meningiomas. Definitive‐intent external beam radiotherapy protocols have historically delivered 18–22 fractions, typically totaling 45–54 Gy, to balance tumour control with late normal‐tissue toxicity [1, 2, 3]. However, the logistic, anaesthetic and economic burden associated with multi‐week treatment courses has prompted investigation into shorter hypofractionated approaches in veterinary radiation oncology [4, 5, 6, 7]. While hypofractionation offers significant advantages in terms of convenience and cost‐effectiveness, careful consideration is required to mitigate potential increases in late toxicity associated with larger per‐fraction doses and shorter treatment time [8]. Improvements in treatment planning and delivery—such as image‐guided radiotherapy (IGRT), intensity‐modulated radiation therapy (IMRT), and techniques including simultaneously integrated boost (SIB) and heterogeneous dose painting—have increased dose conformity and reduced normal tissue exposure, allowing exploration of dose‐escalated hypofractionation with acceptable toxicity. Nevertheless, evaluation of outcome, for example, long‐term tumour control, survival, and radiation‐related side effects remains essential to refine treatment recommendations towards shorter treatment schedules.

In parallel with the use of ultra‐hypofractionated stereotactic radiotherapy protocols for canine meningiomas [4, 5, 7], our institution developed and tested ‘the Zurich Protocol’, a moderately hypofractionated 10‐fraction definitive‐intent protocol for the treatment of canine patients with intracranial meningiomas and other brain tumours. This protocol was designed to accommodate owner preferences for shorter treatment courses and fewer anaesthetic events. Using radiobiological isoeffect calculations, the regimen was planned to maintain tumour control probability equal to conventional 20‐fraction schedules (20 × 2.5Gy), while modelling complication risks for the brain, brainstem, and optic chiasm [2, 8]. Subsequent risk evaluations indicated that doses up to 4.35 Gy per fraction were safe for most patients [8]. In an initial owner‐preference trial, we compared outcomes for 56 dogs with brain tumours treated with either 10 or 20 fractions. For safety, the 10‐fraction group received 4 Gy per fraction rather than the model‐derived 4.35 Gy [6]. Building on these findings, we conducted a randomized clinical trial incorporating an 11% SIB to the gross tumour volume (GTV) to achieve tumour doses more closely equivalent to these of conventional definitive‐intent regimens [9]. Preliminary evidence suggests that 10‐fraction regimens achieve tumour control and survival outcomes comparable to those of smaller‐fractionated definitive‐intent schedules while reducing treatment duration and anaesthetic burden, and enhancing owner compliance and convenience [6, 9].

The objective of the present retrospective study was to evaluate oncologic outcomes, toxicity, and radiation dose‐volume‐related prognostic factors in dogs with imaging‐diagnosed intracranial meningiomas treated with a definitive‐intent 10‐fraction radiotherapy protocol. Outcomes were assessed across different planning approaches, including homogeneous dose prescription, heterogeneous risk‐adapted dose distributions, and SIB. The findings are contextualized relative to published data for conventional 20‐fraction definitive‐intent regimens [1, 2, 3], with the aim of assessing the clinical feasibility, safety, and effectiveness of this shortened moderately hypofractionated regimen.

## 2 Material and Methods

### 2.1 Study Design and Inclusion Criteria

This retrospective observational study included dogs of all breeds, ages, and sexes referred for radiotherapy to the Clinic for Radiation Oncology & Medical Oncology of the University Animal Hospital Zurich between 2015 and 2025, presenting with neurologic signs and imaging‐based diagnosis of an intracranial meningioma. Data from a subset of the dogs included in this cohort have been reported previously [6, 9]. The present analysis includes additional cases, longer follow‐up, and addresses distinct study objectives.

Inclusion criteria required the presence of an extra‐parenchymal, non‐pituitary intracranial mass interpreted as a ‘meningioma’ based on diagnostic imaging, with supportive findings from additional examinations such as cerebrospinal fluid (CSF) analysis when available. Dogs had not undergone prior surgery, radiation therapy, or chemotherapy and showed no evidence of metastatic disease at the time of diagnosis. Initial evaluation included a history and physical examination, documentation of initial neurologic signs at presentation, steroid and other concurrent medical treatments including corticosteroids, brain imaging, and tumour staging with thoracic and abdominal imaging in selected cases when clinically indicated.

### 2.2 Radiation Therapy and Supportive Treatment

Supportive medical treatment before and after radiotherapy was tailored to individual patient needs. Medical treatment was initiated at diagnosis or upon onset of neurologic signs and commonly included corticosteroids and anticonvulsive medications in dogs presenting with seizures.

Dogs were treated with intensity‐modulated radiotherapy (IMRT), or volumetric modulated arc therapy (VMAT) delivered in 10 fractions, with or without a simultaneously integrated boost (SIB), or using heterogeneous, risk‐adapted dose prescriptions. Gross tumour volume (GTV) was contoured on contrast‐enhanced computed tomography (CT) and, when available, magnetic resonance imaging (MRI, T1‐weighted, gadolinium‐enhanced) [10]. The 2 mm clinical target volume (CTV) margin accounted for potential subclinical disease extension, and a margin of 1–2 mm was applied to generate the planning target volume (PTV). Organs at risk (OAR) were delineated as previously described [8]. Treatment planning was performed according to IMRT recommendations [11]. Dose was prescribed in 10 fractions either homogeneously (10 × 4 Gy) [11], with an 11% SIB to the GTV (10 × 4.45 Gy) [9], or as a risk‐adapted, heterogeneous dose prescription [12]. The risk‐adapted, heterogeneous dose prescription was based on a 10 × 4 Gy treatment prescription allowing for heterogeneous dose distribution within the PTV with dose maxima of up to 130%, provided that the constraints for organs at risk could be met [12]. Patients participating in the clinical trials for SIB and heterogeneous dose prescription were assorted randomized to treatment groups. All non clinical‐trial patients were treated with homogeneous dose prescription up to 2022 [9] and with either homogeneous or SIB protocols as chosen by the treating radiation oncologist afterwards. All treatments were delivered using a Varian Clinac iX or Varian TrueBeam linear accelerator with 6MV photons using intensity‐modulated treatment (IMRT or VMAT), with daily image‐guided positioning verification. Treatment was delivered daily (Monday through Friday) over 2 weeks.

### 2.3 Assessment After Therapy and Follow‐Up

Dogs were clinically and neurologically assessed at 3 weeks, 3 months, and every 6 months after radiotherapy until progression or death. Follow‐up imaging was advised every 6 months or at recurrence of neurologic signs. When in‐person evaluation was not feasible, follow‐up was conducted via telephone or electronic communication. Tumour progression was defined by either radiologic evidence of tumour growth or sustained neurologic deterioration not attributable to transient treatment effects and not responsive to corticosteroid therapy. Dogs exhibiting transient or steroid‐responsive neurologic changes were not classified as progressive. Determination of progression was based at the clinical team's discretion, which included neurologic examination findings, diagnostic imaging when available, or necropsy results [13, 14].

Because late radiation‐associated toxicity and tumour progression can produce overlapping neurologic signs in follow‐up, cases without confirmatory imaging or necropsy were conservatively classified as progressive when sustained deterioration was judged unlikely to represent a transient or steroid‐responsive treatment effect.

### 2.4 Statistical Analysis

Data were recorded in Microsoft Excel and analysed using IBM SPSS Statistics (version 31). Data distribution was assessed graphically and using the Shapiro–Wilk test. Descriptive statistics are reported as mean ± standard deviation or median with interquartile range (IQR) or range, as appropriate.

Time to progression (TTP) was defined as the interval from the first day of radiotherapy to clinically or imaging‐confirmed tumour progression. Dogs without progression at the time of analysis, those that died prior to progression, or those lost to follow‐up were censored.

Overall survival after first radiotherapy (OS first RT) was defined as the interval from the first day of radiotherapy to death, or censoring at the start of the second course of radiotherapy when applicable. Dogs still alive without re‐irradiation were censored at the date of the last clinical visit or the most recent contact with the owner or referring veterinarian at which the dog was confirmed to be alive. Overall survival for the entire cohort (OS all) was defined as the interval from the first day of radiotherapy to death from any cause or last follow‐up, including outcomes after a second course of radiotherapy when applicable. Disease‐specific survival (DSS) was defined as the interval from the first day of radiotherapy to death attributable to the disease under investigation or to potential treatment‐related effects. Deaths clearly resulting from unrelated causes, as well as euthanasia for reasons not associated with tumour progression or treatment effects, were classified as censored observations. Survival outcomes were estimated using the Kaplan–Meier method and compared using the log‐rank or Breslow‐Gehan‐Wilcoxon tests as appropriate. For selected prognostic factors, Cox proportional hazards models adjusted for patient weight were fitted for TTP, OS first RT, OS all, and DSS. The prespecified factors reported in Table 3 were age at diagnosis, sex, tumour location, use of a simultaneously integrated boost versus the homogeneous protocol, seizures after radiotherapy, and brain‐GTV V32Gy > 11.5 cm^3^. Because the heterogeneous protocol subgroup was small (*n* = 10), some data were missing, and the resulting complete‐case datasets yielded only 31 to 58 events across endpoints, full multivariable Cox models including all prespecified covariates were considered exploratory and were not used as the primary analysis. Exploratory penalized multivariable models on the complete‐case homogeneous/SIB subset (*n* = 88) were broadly consistent with the main findings. Selection of brain dose‐volume parameters, including V32Gy and generalized equivalent uniform dose, was based on previously established univariate threshold optimization as published: [15] In brief, for each dosimetric metric, thresholds between the 10th and 90th percentiles were tested. Patients were dichotomized at each cutoff, and log‐rank tests were used to assess survival differences. The threshold with the lowest *p* value was selected. Kaplan–Meier curves and univariate Cox models were generated for the optimal cutoff, reporting median survival differences and hazard ratios. Statistical significance was set at *p* < 0.05 (two‐tailed). Survival estimates are reported with corresponding 95% confidence intervals (95% CI). A *p* < 0.05 was considered statistically significant.

## 3 Results

### 3.1 Dog and Tumour Characteristics

One hundred and ten dogs were included, patient and tumour characteristics are presented by treatment groups in Table 1. Median age at diagnosis was 10.0 years (IQR 8.0–11.0 years) and median body weight was 19.7 kg (range 1.9–40.0 kg). Most dogs were of mixed breed, and six dogs (5.5%) were from brachycephalic breeds. Diagnosis was based on MRI in 89 dogs (80.9%) and CT in the remaining cases. Meningioma was histopathologically confirmed in 7 dogs (*n* = 6.4%): in 4 dogs diagnosis was obtained postmortem, and in 3 dogs before treatment via stereotactic biopsy. All dogs presented with neurologic signs. The most common sign was seizures in 54 dogs (49.0%). In all but one dog with seizures, tumours were localized rostrotentorially. Among the 56 dogs without seizures at presentation, tumours were caudotentorial in 42 dogs and rostrotentorial in 14 dogs. As stated above, 40 dogs (44.5%) included in the present analysis were previously reported in studies [6, 9, 15]; however, the results presented here represent novel analyses that have not been reported previously.

**TABLE 1:** Patient and tumour characteristics displayed by treatment groups.

	Total (*n* = 110)	Homogenous [10 × 4 Gy] (*n* = 74)	Heterogenous (*n* = 10)	SIB [10 × 4 Gy + 11% boost to the GTV] (*n* = 26)
Age (years)	9.9 ± 2.1	9.94 ± 2.33	9.5 ± 1.6	9.96 ± 1.77
Weight (kg)	19.7 ± 9.9	19.0 ± 9.5	17.6 ± 9.0	22.3 ± 10.9
Sex				
Male	12 (10.9%)	9 (8.2%)	1 (0.9%)	2 (1.8%)
Male neutered	26 (23.6%)	18 (16.3%)	1 (0.9%)	7 (6.4%)
Female	2 (1.8%)	2 (1.8%)	0	0
Female spayed	70 (63.6%)	45 (40.9%)	8 (7.3%)	17 (15.5%)
Breed				
Pure breed	68 (61.9%)	50 (45.5%)	4 (3.6%)	14 (12.7%)
Mixed breed	42 (38.1%)	24 (21.8%)	6 (5.5%)	12 (10.9%)
Brachycephalic	6 (5.5%)	5 (4.6%)	0	1 (0.9%)
Non‐brachycephalic	104 (94.5%)	69 (62.7%)	10 (9.1%)	25 (22.7%)
Histological tumour verification	7 (6.4%)	3 (2.7%)	0	4 (3.6%)
Tumour location				
Rostrotentorial	67 (60.9%)	39 (35.5%)	7 (6.4%)	21 (19.1%)
Caudotentorial	43 (39.1%)	35 (31.8%)	3 (2.7%)	5 (4.5%)
MRI at diagnosis	89 (80.9%)	61 (55.5%)	9 (8.2%)	19 (17.3%)
Seizures pre—RT	54 (49.1%)	32 (29.1%)	5 (4.5%)	17 (15.5%)
Seizures post—RT				
Yes	27 (24.5%)	14 (12.7%)	3 (2.7%)	10 (9.1%)
Yes, at progression	10 (9.1%)	6 (5.5%)	1 (0.9%)	3 (2.7%)
Corticosteroids pre—RT				
Yes	100 (90.9%)	65 (59.1%)	10 (9.1%)	25 (22.7%)
Dose [mg/kg]	0.7 (0.2–2.0)	0.7 (0.2–2.0)	0.7 (0.5–1.2)	0.65 (0.3–1.3)
Corticosteroids post—RT				
Yes	100 (90.9%)	65 (59.1%)	10 (9.1%)	25 (22.7%)
Dose [mg/kg]	0.6 (0–2.0)	0.6 (0.12–1.9)	0.5 (0–0.9)	0.6 (0.15–1.5)
Anticonvulsive medical treatment				
None	53 (48.2%)	39 (35.5%)	5 (4.5%)	9 (8.2%)
Phenobarbital	18 (16.4%)	11 (10%)	1 (0.9%)	6 (5.5%)
Levetiracetam	9 (8.2%)	7 (6.4%)	0	2 (1.8%)
Levetiracetam and phenobarbital	30 (27.3%)	17 (15.5%)	4 (3.6%)	9 (8.2%)
Continued anticonvulsive treatment post RT	57 (51.8%)	39 (35.5%)	5 (4.5%)	17 (15.5%)
2. RT Series	9 (8.2%)	7 (6.4%)	1 (0.9%)	1 (0.9%)
Dead	76 (69.1%)	52 (47.3%)	7 (6.4%)	17 (15.5%)

### 3.2 Radiation Therapy and Acute Toxicity

All dogs were treated with 10 daily fractions of radiation therapy, with a median overall treatment time of 13 days (range 12–17). Seventy‐four dogs (74/110; 67.3%) were treated with a homogenous treatment protocol. In 10 dogs (10/110; 9.1%), the treatment using a heterogeneous treatment protocol, and 26 dogs (26/110; 23.6%) received a treatment with SIB to the GTV.

**FIGURE 1:**
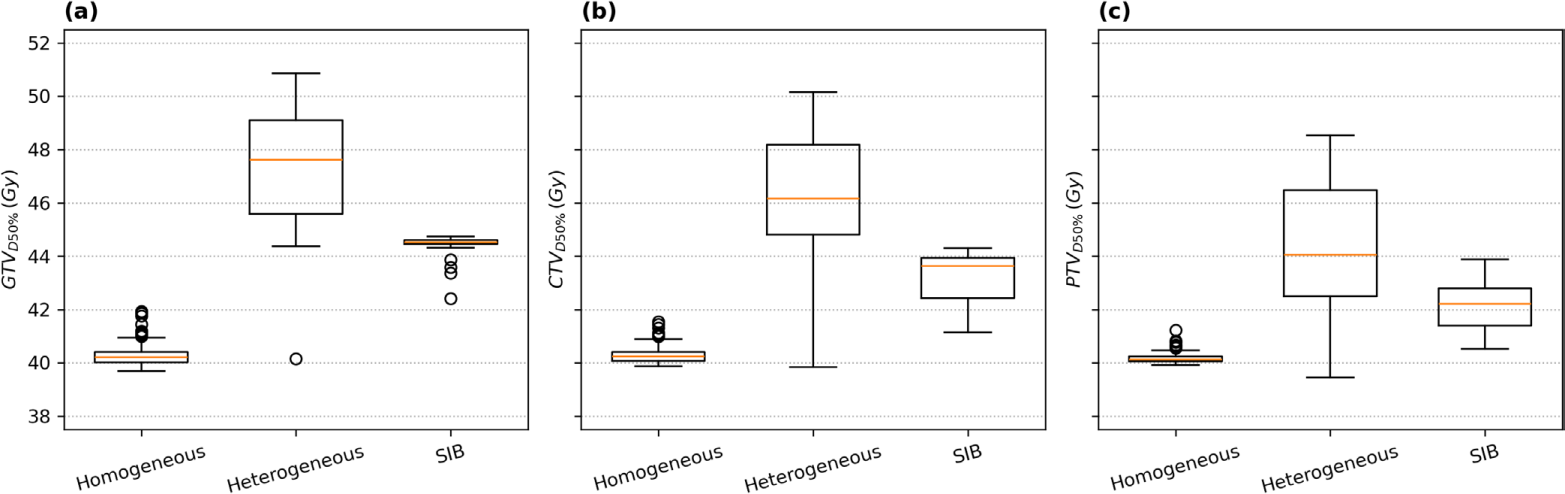
Box plots of D50% for (a) GTVD50%, (b) CTVD50%, and (c) PTVD50% comparing homogeneous, heterogeneous, and SIB groups.

Radiation dose and volume data, reported as mean and SD for the target volumes, are summarized in Table 2 by treatment group. As expected from the planning approach, target D50 distributions differed significantly across the three protocol groups for GTV, CTV, and PTV (all Kruskal‐Wallis *p* < 0.001; Figure 1).

**TABLE 2:** Dose data displayed by treatment groups.

	Total	Homogenous [10 × 4 Gy] (*n* = 74)	Heterogenous (*n* = 10)	SIB [10 × 4 Gy + 11% boost to the GTV] (*n* = 26)	Comparison among groups (0 = homogeneous, 1 = heterogeneous, 2 = SIB)
Volume GTV	2.46 cm^3^ IQR 2.8 (0.15–12.06)	2.66 cm^3^ IQR 2.87 (0.15–12.06)	2.01 cm^3^ IQR 1.16 (1.22–4.82)	2.90 cm^3^ IQR 2.23 (0.67–11.88)	n.s.
Volume CTV	4.98 cm^3^ IQR 4.50 (0.65–67.22)	5.21 cm^3^ IQR 5.28 (0.65–67.22)	4.57 cm^3^ IQR 2.45 (2.46–8.59)	6.49 cm^3^ IQR 5.48 (2.92–28.59)	n.s.
Volume PTV	8.98 cm^3^ IQR 92.49 (1.83–94.32)	9.41 cm^3^ IQR 8.34 (1.83–94.32)	8.03 cm^3^ IQR 3.34 (5.26–14.80)	10.93 cm^3^ IQR 8.82 (3.80–38.02)	n.s.
GTV D98	39.72 Gy IQR 10.29 (38.44–48.74)	39.59 Gy IQR 0.40 (38.44–41.31)	43.67 Gy IQR 8.28 (38.57–48.74)	42.82 Gy IQR 0.88 (40.90–43.57)	*p* = < 0.001 0–1 0–2
GTV D50	40.38 Gy IQR 11.18 (39.86–50.86)	40.17 Gy IQR 0.34 (39.68–41.92)	47.62 Gy IQR 4.64 (40.15–50.85)	44.51 Gy IQR 0.16 (42.41–44.73)	*p* = < 0.001 0–1 0–2
GTV D2	41.25 Gy IQR 11.52 (40.27–51.96)	40.85 Gy IQR 0.66 (40.27–44.18)	50.41 Gy IQR 1.60 (44.95–51.96)	45.52 Gy IQR 0.66 (44.66–47.12)	*p* = < 0.001 0–1 0–2
CTV D98	39.61 Gy IQR 46.99 (0.00–47.0)	39.43 Gy IQR 0.57 (37.27–40.69)	40.78 Gy IQR 8.92 (35.77–47.0)	40.47 Gy IQR 1.28 (0.00–42.08)	*p* = < 0.001 0–2
CTV D50	40.38 Gy IQR 50.15 (0.02–50.16)	40.19 Gy IQR 0.32 (39.87–41.54)	46.16 Gy IQR 5.22 (39.84–50.16)	43.61 Gy IQR 1.70 (0.02–44.3)	*p* = < 0.001 0–1 0–2
CTV D2	41.40 Gy IQR 51.89 (0.04–51.93)	41.01 Gy IQR 0.53 (40.52–43.98)	50.07 Gy IQR 1.69 (45.13–51.92)	45.32 Gy IQR 0.68 (0.04–46.63)	*p* = < 0.001 0–1 0–2
PTV D98	38.29 Gy IQR 14.99 (28.01–43.0)	38.25 Gy IQR 0.61 (36.17–39.34)	37.04 Gy IQR 7.04 (28.01–43.00)	38.30 Gy IQR 0.74 (36.18–39.91)	n.s.
PTV D50	40.20 Gy IQR 9.09 (39.45–48.5)	40.11 Gy IQR 0.16 (39.92–41.22)	44.05 Gy IQR 5.29 (39.45–48.54)	42.16 Gy IQR 1.54 (40.52–43.89)	*p* = < 0.001 0–1 0–2
PTV D2	41.46 Gy IQR 11.06 (40.69–51.76)	41.14 Gy IQR 0.55 (40.69–43.69)	49.83 Gy IQR 2.19 (44.93–51.76)	45.36 Gy IQR 0.60 (43.82–46.50)	*p* = < 0.001 0–1 0–2
Brain‐GTV gEUD	25.03 cm^3^ ± 6.91	23.55 cm^3^ ± 2.67	23.39 cm^3^ ± 3.27	24.04 cm^3^ ± 2.68	n.s.
Brain‐GTV V32Gy	8.67 cm^3^ IQR 19.28 (2.85–22.94)	8.92 cm^3^ IQR 4.41 (2.85–22.94)	6.05 cm^3^ IQR 3.74 (3.75–17.23)	10.18 cm^3^ IQR 6.70 (3.66–21.20)	n.s.
Brain‐GTV V32Gy > 11.5 cm^3^ (available for *n* = 105)	23/105 (21.9%)	12/70 (17.1%)	1/10 (10%)	10/25 (40.0%)	

*Note:* Values are displayed in median, IQR and range, or mean and range respectively.

Acute and early delayed side effects were noted or suspected in 11 dogs (10%). Neurologic signs interpreted as side effects were increased head tilt, gait abnormalities, suspected headache, episodes of imbalance, and coordinative dysfunction. Most of these signs were responsive to a temporary increase of steroids, followed by slower tapering. Four additional dogs experienced an episode of seizures 6–8 weeks following radiotherapy, without preceding neurological signs, and needed seizure‐drug schedule adaptations. One dog, however, presented in status epilepticus and was euthanized. While in the former four dogs these episodes may have been related to subtherapeutic levels of antiepileptic medication or rapid steroid tapering (transient), the dog in status epilepticus succumbed very likely due to radiation‐induced acute effects, such as inflammation and edema [9].

One hundred dogs (100/110; 90.1%) were treated with corticosteroids from the time of diagnosis at a mean dose of 0.7 mg/kg (range 0.2–2.0 mg/kg). In case of seizures, dogs were started on anticonvulsive medical treatment. Fifty‐seven dogs (51.8%) received anticonvulsive medication prior to radiation therapy, either phenobarbital, levetiracetam, or a combination thereof (Table 1).

### 3.3 Follow‐Up, Clinical Outcomes and Late Toxicity

At the time of analysis, 34/110 dogs (30.9%) were still alive. Median follow‐up time for all dogs was 498 days (range, 16–2550 days), and for the dogs still alive (*n* = 34) 597 days (range 64–2550 days). Eight (7.3%) of these dogs were lost to follow‐up (median time of follow‐up 498 days, range 80–1470 days) and censored at this point. Fifty‐four dogs (44%) were considered progressive. Of these, 26 dogs (48%) had follow‐up imaging, which we again reviewed. Increase in tumour volume (as relative to the last available imaging) was confirmed in 22 dogs, with mild to substantial volumetric increases. In 4 dogs, no measurable tumour progression was seen despite neurologic worsening. One of these dogs had severe and one moderate perilesional white matter edema, indicative of late toxicity; the other two were unremarkable on imaging in terms of perilesional findings. Those two dogs had the tumour localized at the brainstem and showed increased central vestibular symptoms. Overall, perilesional edema was mildly increased in several cases with imaging follow‐up, and 2 dogs showed perilesional microbleeds. Necrosis was not listed as radiological differential diagnoses in any of the dogs, but it should be noted that imaging was often not performed at our facility, leading to variability in image quality and scan protocol parameters. The remaining 27 dogs were classified as progressive based on clinical signs, without follow‐up imaging. Death was attributed to the brain tumour or neurologic signs in 44 dogs (40.0%), while 32 dogs (29.1%) died from causes unrelated to the tumour. Unrelated causes of death were categorized as follows: other neoplastic diseases (7 dogs), non‐neoplastic organ‐related conditions (18 dogs), old age (4 dogs), and trauma‐related causes, including accidents and foreign body ingestion (3 dogs). Only 5 of 76 dogs underwent necropsy examination.

### 3.4 Survival Outcomes

Median time to progression was 873 days (95% CI: 552–1195). When split by treatment group, median TTP was 1010 days (95% CI: 776–1245 days) for the 10 × 4 Gy homogeneous treatment protocol, 658 days (95% CI: 435–882 days) for the heterogeneous treatment group and 666 days (95% CI: 26–1307 days) for the SIB group, *p* = 0.024. Median overall survival after the first round of radiation therapy (OS first RT) was 756 days (95% CI: 474–1039 days), with 1‐, 2‐, and 3‐year overall survival rates of 66% (95% CI: 57%–75%), 50% (95% CI: 39%–60%) and 32% (95% CI: 21%–43%). For the homogeneous treatment group, the median OS after first treatment was 814 days (95% CI: 380–1249 days), 713 days (95% CI: 420–1007 days) for the heterogeneous group and 305 days (0–1003 days) for the SIB group, n.s., *p* = 0.06.

### 3.5 Prognostic Factors and Dose‐Volume Analysis

Multiple prognostic factors were identified in the weight‐adjusted models. Weight‐adjusted Cox proportional hazard models for the selected prognostic factors are summarized in Table 3.

**TABLE 3:** Cox proportional hazards regression adjusted for patient weight for prognostic factors.

	TTP (HR; 95% CI; *p*)	OS after first RT (HR; 95% CI; *p*)	OS all* (HR; 95% CI; *p*)	DSS (HR; 95% CI; *p*)
Age	1.23 (1.08–1.41; *p* = 0.002)	1.19 (1.06–1.33; *p* = 0.003)	1.14 (1.03–1.27; *p* = 0.013)	1.14 (0.99–1.31; *p* = 0.070)
Other versus male intact	0.88 (0.37–2.08; *p* = 0.766)	0.71 (0.34–1.52; *p* = 0.381)	0.66 (0.32–1.35; *p* = 0.253)	0.43 (0.19–0.96; *p* = 0.039)
Homogeneous versus boost protocol^**^	2.38 (1.25–4.54; *p* = 0.008)	2.20 (1.20–4.00; *p* = 0.010)	1.89 (1.07–3.35; *p* = 0.029)	2.41 (1.19–4.89; *p* = 0.014)
Brain/dose V32Gy > 11.5 cm3	3.17 (1.77–5.69; *p* = 0.000)	2.51 (1.46–4.29; *p* = 0.001)	2.34 (1.38–3.97; *p* = 0.002)	3.07 (1.59–5.92; *p* = 0.001)
Seizures post radiation	1.94 (1.05–3.57; *p* = 0.034)	1.22 (0.70–2.12; *p* = 0.475)	1.05 (0.61–1.81; *p* = 0.854)	1.05 (0.61–1.81; *p* = 0.854)
Caudotentorial tumour location versus rostrotentorial tumour location	0.88 (0.52–1.52; *p* = 0.654)	0.82 (0.51–1.34; *p* = 0.429)	0.81 (0.51–1.28; *p* = 0.371)	0.67 (0.36–1.24; *p* = 0.201)

* (Including dogs that underwent a second course of radiation).

** No difference was seen among treatment groups other than the ones displayed here.

Use of an SIB versus the homogeneous protocol was associated with shorter median TTP, OS first RT, OS all, and DSS (Table 3). Larger brain‐GTV V32Gy (> 11.5 cm3) was likewise associated with shorter outcomes across all endpoints. Older age at diagnosis was negatively associated with all endpoints, whereas post‐radiotherapy seizures were associated with shorter TTP only. No significant difference was detected between rostrotentorial and caudotentorial tumour location. Additional weight‐adjusted pairwise protocol comparisons did not identify significant differences for heterogeneous versus homogeneous or SIB versus heterogeneous for any endpoint, although these comparisons were limited by the small heterogeneous subgroup (*n* = 10). Figure 1 illustrates the intended target‐dose separation between protocols, whereas the clinically relevant protocol‐outcome associations are summarized in Table 3.

**FIGURE 2:**
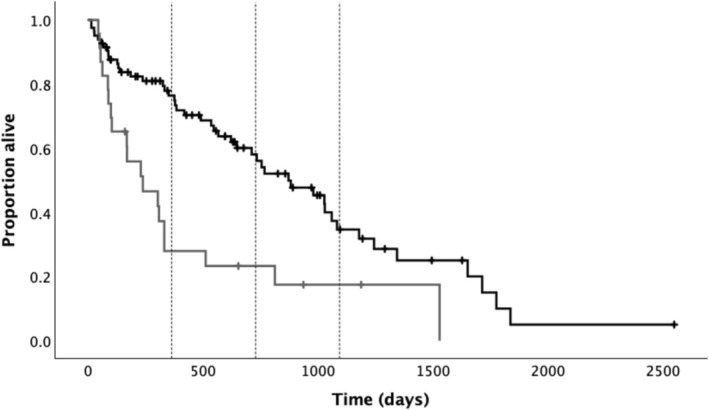
Kaplan–Meier survival curves for the selected dose–volume threshold brain–GTV V32 Gy > 11.5 versus ≤ 11.5 cm^3^: Grey line—V32Gy > 11.5 cm^3^—median survival of 240 days (95% CI: 41–440 days); black line—constraint of brain‐GTV V32Gy ≤ 11.5 cm^3^—with median survival of 873 days (95% CI: 594–1153 days), *p* < 0.001. The tick marks represent censored cases, the vertical dotted lines mark 1, 2 and 3 years.

Of the 23 dogs with irradiated brain‐GTV volume above 11.5cm^3^,12 had imaging performed either at progression or as a regular recheck. In none of the imaging studies necrosis was suspected. When the dogs were split by the identified cutoff‐constraint of brain volume at high dose outside the GTV (brain‐GTV), overall survival significantly differed: the dogs within the constraint of brain‐GTV (V32Gy ≤ 11.5 cm^3^, *n* = 82) had a much longer median survival of 873 days (95% CI: 594–1153 days), whereas dogs with brain‐GTV V32Gy > 11.5 cm^3^, *n* = 23, had a median survival of 240 days (95% CI: 41–440 days), *p* < 0.001 (Figure 2). As shown in Table 2, in 40% of the dogs in the SIB group exceeded this later‐identified constraint.

Overall median DSS was 1078 days (95% CI: 915–1242 days), with 1‐, 2‐, and 3‐year DSS rates of 80% (95% CI: 72%–88%), 68% (95% CI: 57%–79%), and 46% (95% CI: 33%–59%). For the homogeneous treatment protocol, the median DSS was 1115 days (95% CI: 971–1260 days) and 1386 days (95% CI: 380–2393 days) for the heterogeneous protocol. For the SIB group, the median DSS was 873 days (95% CI: 0–1799 days), *p* = 0.015.

Nine dogs were treated with a second course of radiation therapy, delivered in 10 daily fractions of 4 Gy. Seven of these dogs have previously been treated with the homogeneous protocol, while one dog was treated with the heterogeneous protocol and one dog was treated with the SIB protocol in their first treatment course. Local tumour control and remission of clinical signs were again achieved for a median of 384 days (95% CI: 10–759 days, range 127–708 days). One dog was still alive at time of reporting, and 2 dogs were lost to follow up after their second treatment course.

When follow‐up after the second course of radiation therapy was included (OS all), the median overall survival for the entire cohort was also 756 days (95% CI: 480–1033 days). The corresponding 1‐, 2‐, and 3‐year OS were 67% (95% CI: 57–75), 52% (95% CI: 41–61), and 31% (95% CI: 22–42), respectively. The 1‐, 2‐, and 3‐year DSS rates were 81% (95% CI: 72–88), 70% (95% CI: 58–78), and 48% (95% CI: 35–60), respectively.

## 4 Discussion

In this retrospective cohort, the definitive‐intent 10‐fraction radiotherapy protocol was associated with durable tumour control, encouraging survival outcomes, and acceptable toxicity profiles comparable to those reported for conventional 20‐fraction definitive‐intent regimens in dogs with imaging‐diagnosed meningiomas. One‐, two‐, and three‐year overall survival rates of 66%, 50%, and 32% were similar to previously published overall survival rates of 70%, 45%, and 18%, reported for conventional 20‐fraction definitive‐intent regimens in dogs with imaging‐diagnosed meningiomas [2]. The high proportion of dogs with 1‐, 2‐, and 3‐year DSS rates of 80%, 68%, and 46%, again comparable to the disease‐specific median of 2.5 years found prior [2] which suggests that a substantial proportion of these dogs died from causes unrelated to their intracranial disease. However, as no direct control group was included and treatments were performed at different institutions, such cross‐study comparisons should be interpreted cautiously.

Older age was identified as a mild negative prognostic factor and might reflect greater fragility of older patients and their brain tissue. Persistence of seizures post radiotherapy was associated with an approximately twofold higher hazard of shorter time to (presumed) progression. This is consistent with established evidence that dogs treated with radiotherapy are much longer seizure free than dogs treated with antiepileptic drugs alone [16]. There is no robust evidence that dogs with brain tumours post‐radiotherapy consistently require higher doses of antiepileptic medication compared to dogs with epilepsy from other causes. In a prior study of our group, however, we found significantly lower serum phenobarbital levels in patients with post‐radiation uncontrolled seizures than in dogs with well controlled epilepsy (16.4 vs. 25.4 μg/mL) [9]. Meningiomas often regress slowly after radiotherapy, potentially maintaining an epileptogenic stimulus despite (volumetric) tumour control. In humans, persistent epilepsy after complete surgical excision has been reported in 19% of patients with meningioma, possibly related in part to the surgical intervention itself [17]. Radiation therapy has not been associated with refractory seizures in human meningiomas patients [18].

Clinically apparent acute or early‐delayed radiation‐induced toxicity was mostly transient and occurred in a small proportion of patients. Among the 11 dogs that developed steroid‐responsive neurologic deterioration, or the 5 dogs that showed re‐occurrence of seizures, it is plausible that clinical signs were at least partly related to overly rapid tapering of corticosteroids, highlighting the delicate balance required when managing steroid dose reductions in this population [19]. However, one dog succumbed to status epilepticus 64 days after treatment.

An important consideration in this study relates to the definition of disease progression versus late toxicity. Progression was defined using a combination of imaging findings and sustained neurologic deterioration not responsive to corticosteroid therapy, reflecting standard clinical practice in veterinary neuro‐oncology, and remaining a limitation of study design. Only half of the dogs classified as progressive underwent follow‐up imaging. While in none of the imaging studies necrosis was suspected, it can still not be excluded. Further, it can be assumed that some of the non‐re‐imaged patients also had signs of late adverse radiation‐associated effects such as white matter edema or bleedings. However, such effects were also noted on imaging in dogs not considered progressive and might remain clinically silent. Therefore, follow‐up imaging, even with advanced imaging such as perfusion‐, diffusion‐weighted or MR spectroscopy, cannot be considered confirmative of late toxicity [20]. Seven dogs classified as progressive in the absence of imaging confirmation were above the identified dose‐volume threshold. Two of these died or were euthanized early, within several weeks, in a status epilepticus and other neurologic symptoms, indicative of acute toxicity. The other five were indeed at higher risk of late toxicity. Nevertheless, in two dogs, death was not directly attributed to the brain tumour (but to a post‐operative pneumonia and a bleeding splenic lesion, respectively). Treating with fewer but larger fractions (10 × 4 Gy) could carry a risk for increased late toxicity; however, the total dose of the protocol is lower, at least partially mitigating this risk. Previous work from our group identified an odds ratio of 4.7 for the development of cerebral microbleeds within high‐dose regions exceeding 30 Gy following radiotherapy with this 10‐fraction protocol [21]. Therein, cerebral microbleeds were detected in 62% of dogs post‐radiotherapy, with a time‐dependent increase in prevalence. These microbleeds, however, were not associated with overt neurologic deficits attributable specifically to their presence [21]. While they may be more frequently seen in patients post radiotherapy, their clinical relevance remains unclear and they may not be indicative of radiation toxicity or clinical outcome.

Treatment with the SIB protocol was associated with shorter survival outcomes relative to the homogeneous protocol in the weight‐adjusted models. One possible explanation is that SIB planning raises dose to the entire GTV and may therefore increase high‐dose exposure to adjacent normal tissues if no target compromise is accepted. In contrast, the heterogeneous risk‐adapted approach permits higher intratumoral hot spots while potentially limiting the global dose to surrounding tissues. No significant pairwise difference involving the heterogeneous protocol was identified, but those analyses were based on only 10 dogs and should therefore be interpreted cautiously.

In the current study, brain‐GTV volumes exceeding 11.5 cm^3^ receiving 32 Gy or more, a threshold established in previous investigations of our group, were associated with significantly reduced survival, and this association remained evident in models adjusted for patient weight. Although overt radiation necrosis was not identified in any patient based on follow‐up imaging, imaging follow‐up was incomplete and not standardized, so subtle or non‐classic late normal tissue effects may have been underrecognized. These findings therefore suggest that high‐dose irradiation of normal brain tissue may contribute to increased mortality through subtle, difficult‐to‐detect mechanisms. In particular, radiation‐induced vascular injury at the microstructural level may play a role, even in the absence of classic imaging features of radiation necrosis [15]. For these reasons, a cautious approach to high‐dose exposure of normal brain tissue appears warranted.

On the positive side, these data support the proactive future use of evidence‐based dose‐volume guidelines for the 10‐fraction canine meningioma radiotherapy protocol. Specifically, they reinforce the importance of incorporating brain dose‐volume constraints into treatment planning and minimizing overall brain dose, including adherence to a whole‐brain gEUD of < 30 Gy, as previously recommended [15]. The slight differences in brain‐GTV‐constraint volumes such as V32Gy of < 13 cm^3^ in Unterkirhers et al. 2025 [15] or < 11.5 cm^3^ as found herein are of minor concern, as for safety reasons the lower‐volume constraint should be considered for all tumours. When these constraints are respected, the 10 × 4 Gy protocol—with selective dose escalation where feasible—represents an effective and clinically highly attractive alternative to longer and more costly definitive‐intent fractionated radiotherapy protocols for dogs with meningioma.

While we see a clinical advantage of the shortened, moderately hypofractionated regimen for definitive‐intent radiotherapy of meningiomas in dogs, there is also a radiobiological rationale for treating meningioma with such a hypofractionated regimen. Meningiomas are generally slow‐growing, low‐proliferation tumours with a low α/β‐ratio (estimated around 3 Gy), which means they are more sensitive to changes in fraction size and may respond favourably to larger fraction sizes (hypofractionation) in terms of tumour control [22]. Hypofractionated regimens can deliver a higher biologically effective dose (BED) to the tumour while potentially sparing adjacent normal tissues when delivered with highly conformal techniques.

A limitation of this study is that case inclusion and treatment decisions were based on imaging findings. Although specific imaging features have been shown to correlate with histopathologic diagnoses and may support a presumptive tumour classification [23] histopathology remains the diagnostic gold standard. This limitation likewise applies to the distinction between tumour progression and (late) adverse radiation effects, specifically, as follow‐up imaging at the time of neurologic decline was not consistently available. This limited our ability to find indications between overt tumour progression or possible fatal late toxicity [24]. Potential misclassification may therefore have confounded outcome assessment.

## 5 Conclusion

In conclusion, the definitive‐intent, moderately hypofractionated 10 × 4 Gy radiotherapy protocol achieves durable tumour control and favourable survival outcomes in dogs with imaging‐based meningioma. When evidence‐based brain dose‐volume constraints are respected, this protocol represents a safe, effective, and clinically attractive alternative that reduces treatment duration and treatment burden with excellent oncologic outcomes.

## Funding

The authors have nothing to report.

## Disclosure

Cell line validation statement: No cell lines were used for this work.

## Ethics Statement

Ethical approval for the not previously published additional cases was not required, but written owner consent for anonymous data usage was obtained.

## Conflicts of Interest

The authors declare no conflicts of interest.

## Data Availability

The data that support the findings of this study are available from the corresponding author upon reasonable request.
